# Effect of free gingival graft before implant placement on peri-implant health and soft tissue changes: a randomized controlled trial

**DOI:** 10.1186/s12903-021-01818-3

**Published:** 2021-10-04

**Authors:** Chaoling Zheng, Shimin Wang, Hongqiang Ye, Yunsong Liu, Wenjie Hu, Yongsheng Zhou

**Affiliations:** 1 Department of Prosthodontics, Peking University School and Hospital of Stomatology, Beijing, 100081 People’s Republic of China; 2 National Laboratory for Digital and Material Technology of Stomatology, National Clinical Research Center for Oral Diseases, Beijing Key Laboratory of Digital Stomatology, Beijing, 100081 People’s Republic of China; 3 Department of Periodontics, Peking University School and Hospital of Stomatology, Beijing, 100081 People’s Republic of China

**Keywords:** Posterior implant restoration, Free gingival graft, Soft tissue changes, Clinical outcome

## Abstract

**Background:**

To evaluate the clinical outcome and changes in posterior buccal soft tissue following implant restoration in groups with and without a free gingival graft (FGG) before implant placement.

**Methods:**

Twenty-six individuals who required implant restoration and displayed lack of keratinized mucosa (KM) were recruited and assigned to the FGG group (with FGG before implant placement) or Control group (without FGG before implant placement) randomly. A screw-retained conventional implant restoration was performed for each patient. Peri-implant soft tissue was captured by an intraoral scanner and analyzed by an image processing software. Clinical parameters (plaque index, gingival index, probing depth, and bleeding on probing) were assessed at baseline and 1, 3, 6, and 12 months. Buccal soft tissue changes (mucosal margin, soft tissue thickness, and width of keratinized mucosa) on the buccal side of implant site were assessed at 1, 3, 6, and 12 months. Two-way ANOVA and Bonferroni test were used to analyze significant difference between groups at each time point (α = 0.05).

**Results:**

The clinical parameters were lower in the FGG group than that in the Control group, although there were no significant differences between the two groups (*P* > 0.05). Peri-implant soft tissue collapsed and the changes (mucosal margin and soft tissue thickness) were significantly greater in the Control group than the FGG group (*P* < 0.05). Width of KM was larger in the FGG group than the Control group, although there was no significant difference between the two groups (*P* > 0.05).

**Conclusions:**

Minimal peri-implant soft tissue changes occurred in two groups. Performing FGG before implant placement is a viable procedure to maintain peri-implant soft tissue but might not affect peri-implant health during 12 months follow-up. However, small sample size must be considered.

*Trial registration* This study was retrospectively registered in the Chinese Clinical Trial Registry (Registration number: ChiCTR2000037954; Date of registration: 6 September 2020).

## Background

In recent years, implant-supported dental prostheses have become widely used in patients with a partial edentulous or edentulous jaw, with a high rate of implant survival [1]. The incidence rate of complications (mechanical and biological, et al.) is increasing as implants continuous to be used. Biological complications are also called peri-implant diseases [2] which include peri-implant mucositis and peri-implantitis [3, 4]. Peri-implant diseases are inflammation of peri-implant tissues [5] whose maintenance is a challenge to dentists and patients.

Lack of keratinized mucosa (KM) is a potential risk to peri-implant diseases [6]. Although the role of width of KM in maintaining peri-implant health is a controversial topic [7], several studies showed that peri-implant state and plaque control with adequate KM was better than implant lacking KM [8, 9]. It has been reported that at least 2 mm of KM around implant is positive to maintain peri-implant health [10] and implant with < 2 mm is more vulnerable to peri-implant diseases [11].

Free gingival graft (FGG) is considered as the common approach for increasing KM and soft tissue thickness at natural teeth and implant sites [12]. A systematic review concluded that soft tissue augmentation can be performed before or during implantation, or even after implant restoration if complications occur [13]. One prospective study suggested that FGG for implant site with mucosal recession after restoration is a viable choice to reduce peri-implant inflammation and to maintain crestal bone level during 18 months follow-up [8]. An animal study, which investigated the effect of performing FGG simultaneously with implant placement, found that FGG may reduce the resorption of crestal bone and mucosal recession during 3 months follow-up [14]. Until now, to the best of our knowledge, effect of performing FGG before implant placement is limited to case report which suggested soft tissue growth within 3 months [15]. However, there is no animal or clinical study investigating the effect of this technique on peri-implant tissue after restoration.

Marginal bone level is the common indicator of evaluation for soft tissue augmentation. Remolding process exists in alveolar bone all the time. So does the overlying soft tissue. Periodontal probes and endodontic files are commonly used to evaluate soft tissue changes. At present, digital methods for evaluating peri-implant soft tissue changes have gradually become an outcome of interest in different literatures possibly because of its noninvasiveness [16], accuracy [17], and the digital information could be saved in computer permanently. A recent study summarized the available evidence regarding traditional and digital methods for the evaluation of peri-implant soft tissue changes and concluded that traditional methods can’t show the entire changes while digital methods can, and digital analysis might become the gold standard technique in the future [18]. Several studies have used digital methods to evaluate the changes in peri-implant soft tissue (mucosal recession and soft tissue thickness) after soft tissue augmentation (subepithelial connective tissue graft) [19–21]. However, to the best of our knowledge, there is no study evaluating change in peri-implant soft tissue after FGG before implant placement using digital methods.

The objective of this study, therefore, was to evaluate the clinical outcomes and changes in peri-implant soft tissue during 12 months follow-up in groups with and without a FGG before implant placement. The null hypothesis was that FGG before implant placement would be beneficial to peri-implant health and maintenance of peri-implant soft tissue during the course of this study.

## Methods

This randomized controlled trial aimed to evaluate peri-implant health and soft tissue changes following implant restoration in groups with and without a FGG before implant placement. Clinical parameters were recorded and changes in soft tissue were evaluated using digital method at 1, 3, 6, and 12 months after baseline (two weeks after definitive crown insertion [21, 22]), respectively. The study protocol was carried out in accordance with the Declaration of Helsinki and was approved by the Biomedical Ethics Committee of Peking University Hospital of Stomatology, Beijing, China (ethical batch number: PKUSSIRB-201946083), as well as registered on Chinese Clinical Trial Registry (ChiCTR2000037954). The study was performed according to the CONSORT checklist.

### Subjects

The study was carried out in the Department of Periodontology and Prosthodontics, Peking University School and Hospital of Stomatology, from October 2018 to February 2020. All subjects signed consent forms prior to participation. Continuous patients who required implant restoration were recruited according to the following inclusion criteria: (1) age ≥ 20 years old [23]; (2) no system disease or active periodontitis; (3) plaque index and bleeding index < 25%; (4) posterior teeth loss with a medium to thick gingival biotype (periodontal probe not visible when inserted into the buccal gingival margin) [24]; (5) Width of KM on the buccal side of the implant site < 2 mm (distance from central point of implant site alveolar ridge crest to mucogingival junction [25]) before implant placement; and (6) fully autonomous behavior and expression ability, with good compliance. Exclusion criteria were as follows: (1) poor oral hygiene; (2) adjacent teeth with acute and chronic tooth disease at the implant site; (3) uncontrolled diabetes or other systemic disease; and (4) severe smoker (≥ 10 cigarettes/day). In addition, implants were excluded if they emerged complications during the period of this study that additional treatments were needed.

### Randomization and blinding

This was a complete randomized controlled trial with parallel design (1:1 allocation ratio). The subjects were randomly assigned to either the FGG group or Control group by a dentist who was not involve in this study. A random number sequence was generated by a statistician before recruitment. Allocations were concealed by opaque sealed envelopes. The dentist who enrolled the subjects and the investigator who evaluated peri-implant soft tissue changes were blinded to the study protocol and purpose.

### Surgical procedures

**Fig. 1:**
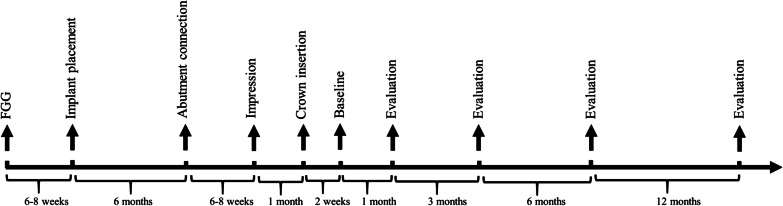
Timeline of clinical treatment and the evaluations of FGG group

All participants underwent systematic supportive periodontal therapy before surgery, and the clinical treatment plan was discussed and agreed prior to implantation. A timeline of the clinical treatment process is shown in Fig. 1.

**Fig. 2:**
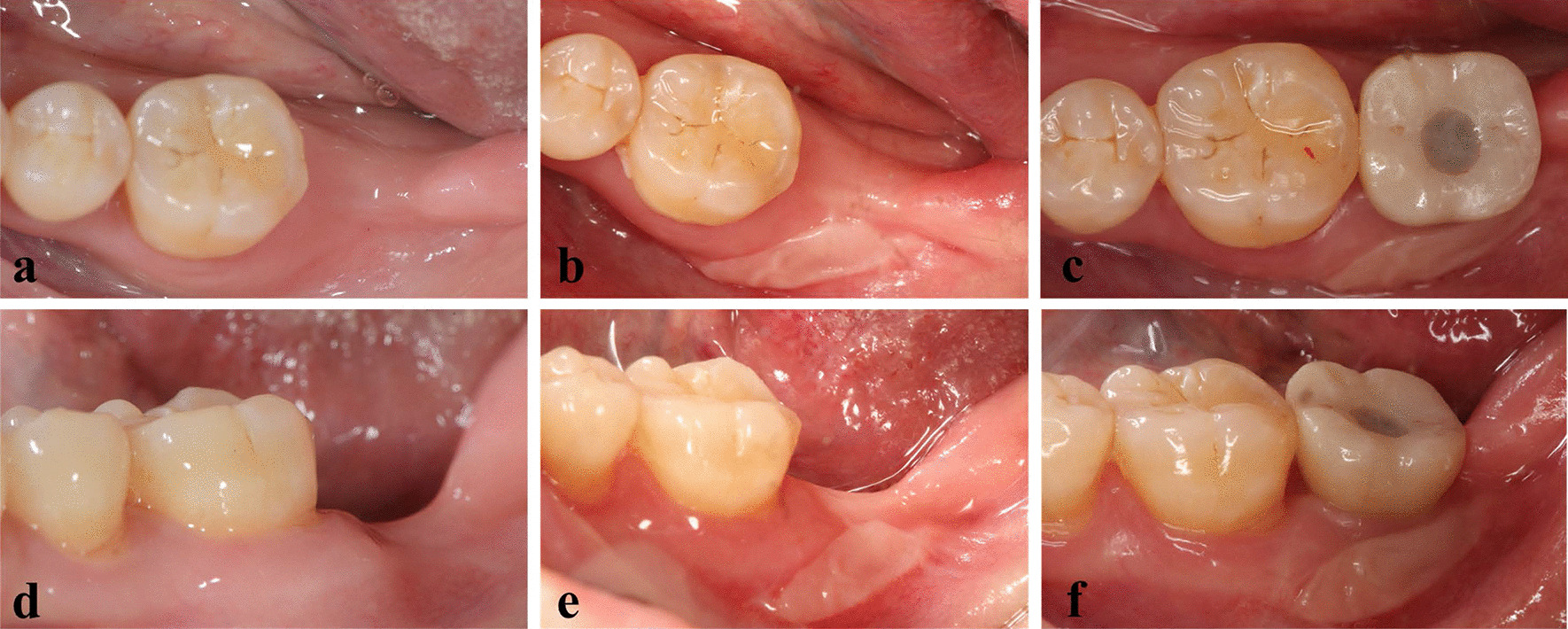
Intraoral characteristics of the FGG group. **a–c** Occlusal and **d–f** buccal views of the implant site before and after placement of the free gingival graft (FGG), and following crown insertion, respectively

Patients in the FGG group underwent a free gingival graft before implant placement. FGGs were harvested from the palate. The width and length of FGGs were 5–8 mm and 10–13 mm, respectively in this study, which were determined by the size of recipient bed. The thickness of FGGs was 1.0–1.5 mm, depending on the amount of tissue available in each patient’s palate. After a healing time of 6–8 weeks, the full-thickness flap was elevated under local anesthesia then the two-stage implant was placed according to the manufacturer’s instructions (Bone Level, Straumann®, Switzerland). A healing abutment was inserted 6 months later followed by a definitive all-ceramic zirconia crown (titanium abutment) 3 months after the second-stage procedure. Patients in the Control group did not undergo FGG before implantation, but all other procedures were the same. Intraoral characteristics of the FGG group are shown in Fig. 2.

### Clinical examination

All subjects received oral prophylaxis. An evaluator recorded the following indicators three times and then averaged. Values were recorded at mesial, midfacial, distal, and palatal sites and then averaged. The implant survival rate was also recorded at baseline and follow-up. Plaque index (PI) [26] and Gingival index (GI) [27] was recorded at implant site. Probing depth (PD) was measured using a manual probe (Williams, Hu-Friedy, Chicago, USA). PD was recorded to the nearest 0.5 mm. Bleeding on probing (BOP, positive or negative; %) was recorded after probing.

### Peri-implant soft tissue evaluations

All measurements were carried out by another evaluator at 1, 3, 6, and 12 months. The evaluator was blind to the study protocol and purpose. The following indicators were recorded three times and then averaged.

#### Intraoral scanning

Before all measurements performed, three-dimensional oral information was obtained using an intraoral scanner (TRIOS Color Pod; software version: 1.18.1.3; 3Shape, Denmark) at each time point. All scanning data were exported into DCM (a software-dedicated file format) format. Then imported into orthodontic software (Ortho Analyzer™, software version: 1.18.1.2, 3Shape, Denmark) and saved as a virtual reality modeling language (VRML) file. A color digital model (colored by VRML procedure) was then obtained and imported into 3D analysis software (Geomagic Control 2014, 3D Systems, Rock Hill, South Carolina, USA).

#### Digital model alignment

**Fig. 3:**
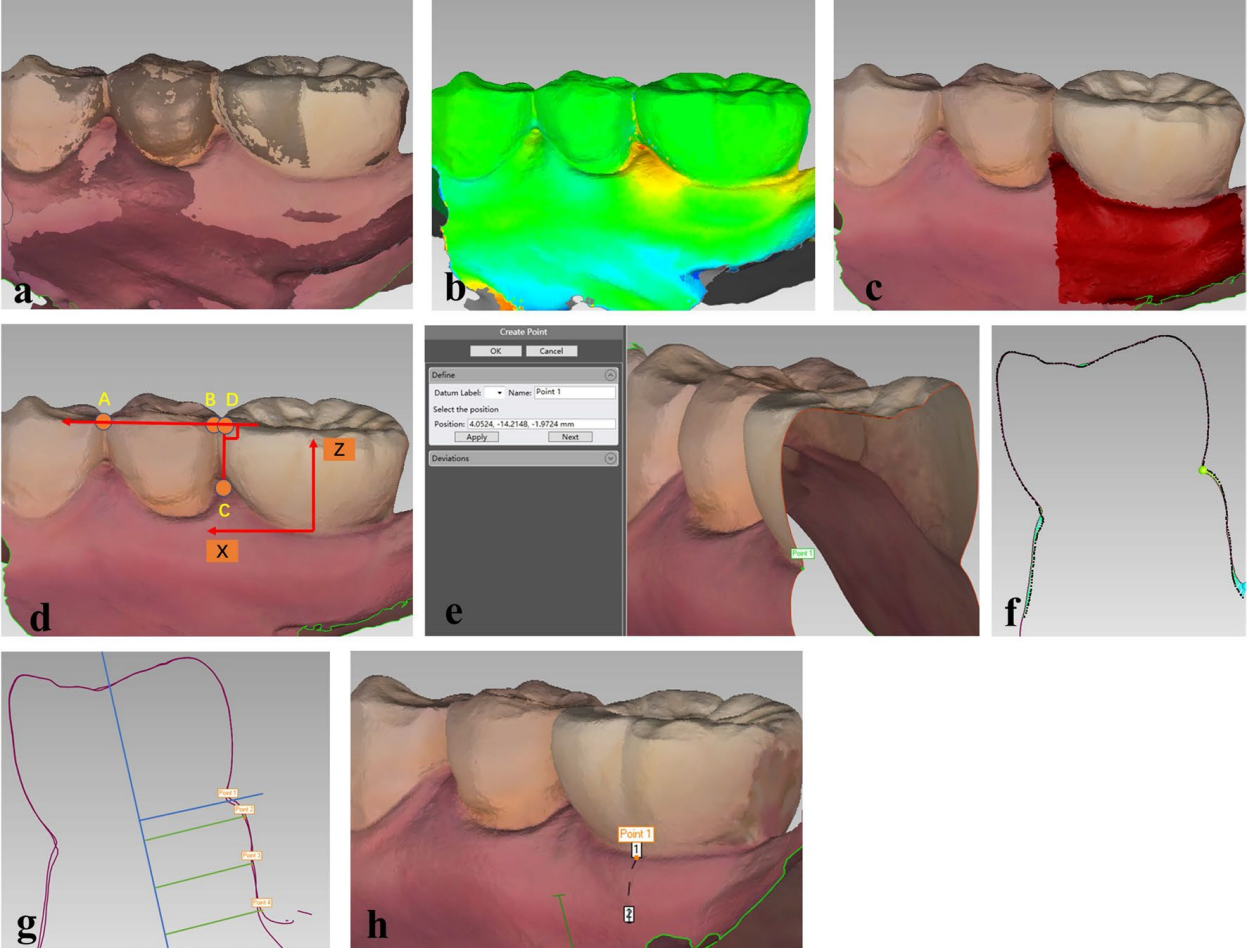
Peri-implant soft tissue evaluation. **a** Registration of the digital model. **b** Three-dimensional deviation analysis. **c** Selection of the area of interest. **d** Establishment of the three-dimensional coordinate system. **e** Selection and recording of coordinates of the mucosal margin. **f** A two-dimensional cross section, and measurements of **g** the changes in soft tissue thickness (ΔASTT) at 1, 3, and 5 mm below the mucosal margin. **h** width of keratinized mucosa (KM)

In the Geomagic Control software, the baseline scanning data was set as the reference model and the follow-up scanning data as the test model, then “best fit alignment” (iterative closet point algorithm) was conducted based on the implant-supported dental prosthesis and adjacent teeth (Fig. 3a). Three-dimensional deviation analysis between the reference model and the test model was also performed using the “3D comparison” function (Fig. 3b). Based on the accuracy of the intraoral scanner, maximum and minimum nominal values were set at 150 and − 150 µm, respectively [28]. The area of interest on the buccal side was then selected (Fig. 3c), with the coronal border represented by the mucosal margin and the apical border by the vestibular groove.

#### Coordinate system establishment

A three-dimensional coordinate system was then established based on the reference model using the "coordinate system" function. Line AB was used as the X-axis (Fig. 3d), with points A and B representing the most coronally adjacent points between the implant and adjacent teeth (if present) or between the two consecutive teeth in front of the implant (if distal adjacent teeth were absent). A vertical line was then drawn from the tip of the mesial papillae gingiva of the implant-supported dental prosthesis (Point C) to line AB, with the base of the perpendicular line representing Point D. The direction of line CD was used as the Z-axis, with the Y-axis running in the buccal/palate direction (to the missing tooth in the upper jaw) or buccal/lingual direction (to the missing tooth in the lower jaw). The positive direction of the X-axis ran from the far side to the midfacial region, while the positive Y-axis ran from the buccal side to the palatal or lingual side, and the positive Z-axis ran from the root to the crown side (Fig. 3d). The established coordinate system was then used as the measurement coordinate system.

#### Analysis of changes in mucosal margin

To examine changes in the mucosal margin (MM), the gingival zenith was first selected then three-dimensional coordinates at mesial, midfacial, and distal sites of the buccal side of the implant-supported dental prosthesis were respectively recorded in the reference model and test model using the "insertion point" function in the Geomagic software (Fig. 3e). The difference between the Z-axis coordinates in the two models was then recorded as the changes in the MM. Changes at the mesial, midfacial, and distal sites were then averaged (ΔAMM). Negative values represent the mucosal recession.

#### Analysis of changes in soft tissue thickness

To examine changes in soft tissue thickness (STT), three two-dimensional cross sections were respectively generated at mesial, midfacial, and distal sites in the registered model using the "2D comparison" function (Fig. 3f). Using the "section position" function within this, it was ensured that the mucosal margin of the three sites was included in the two-dimensional sections of the three sites, respectively. The "distance" function was then selected and "on surface projection" was checked to generate a surface distance of 1, 3, and 5 mm below the mucosal margin at mesial, midfacial, and distal sites of the buccal side in the registered model. To evaluate STT at each level, longitudinal lines were drawn parallel to the long axis of the implant in each two-dimensional section. Distances at 1, 3, and 5 mm levels between the reference model and test model were then used to represent the changes in STT (Fig. 3g). Changes at the three sites were then averaged (ΔASTT). Negative values represent soft tissue collapse.

#### Analysis of changes in width of keratinized mucosa

To examine the changes in width of KM, the mucosal margin to the mucogingival junction at mesial, midfacial and distal sites on the buccal side of the implant were measured using the "distance" function (Fig. 3h). Width of KM values at the three sites were then averaged. Difference between width of KM_baseline_ and KM_Follow-up_ was then used to present the changes in width of KM. Negative values represent reduction in KM.

### Sample size calculation and Statistical Analysis

#### Sample size calculation

Based on the ΔAMM data (the first three patients recruited) of this study, the sample size was calculated using PASS (α = 0.05, 80% power). After calculating, a study population of at least 24 patients was deemed necessary, with 12 patients per group. Considering a dropout rate of 10%, a total of 28 patients were recruited.

#### Statistical analysis

The subject was treated as the unit of statistical analysis. As such, every subject presented with a single implant. SPSS software (IBM®, SPSS®, Statistics 20, USA) was used for data analysis. Descriptive statistics (mean ± SD), normality (Kolmogorov–Smirnov test), and homogeneity of variance (Levene test) were performed for all variables (repeated measurement data). The two-way Analysis of Variance (ANOVA) was used to compare PD, ΔAMM, ΔASTT, and changes in width of KM between the two groups. If proven to be statistically significant, the Bonferroni test was used to perform multiple comparison. The Friedman’s two-way ANOVA by ranks was used to compare PI, GI, and BOP between the two groups. *P* < 0.05 was considered statistically significant.

## Results

### Patients’ and implants’ characteristics

**Fig. 4:**
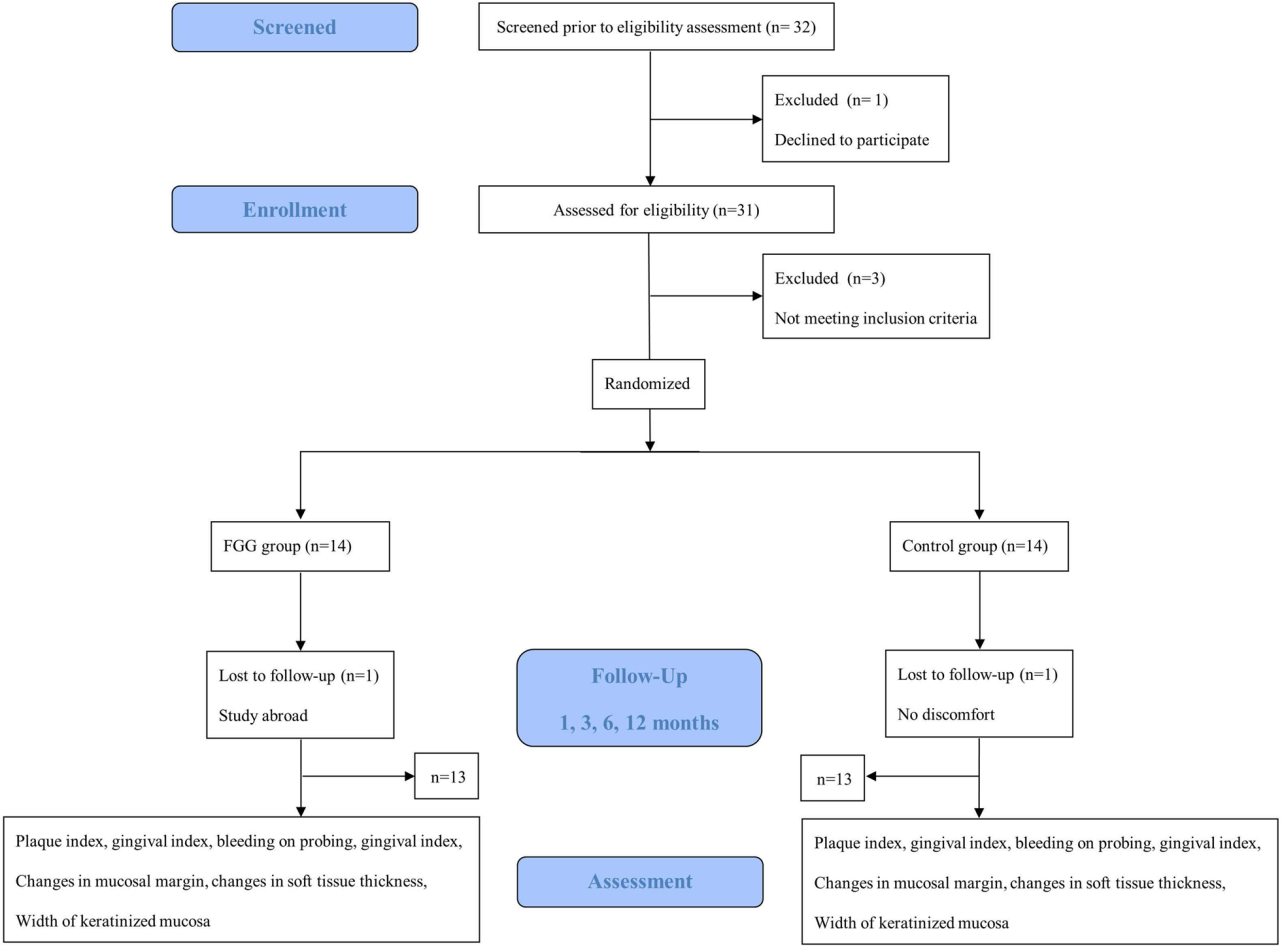
A flow diagram of the recruitment process

A CONSORT flow diagram of the recruitment process is shown in Fig. 4. Of the 28 patients (28 implants) enrolled, two did not complete the follow-up. The one from FGG group study abroad. The one from Control group has no discomfortable. A total of 26 patients (26 implants) were therefore included in this study, 14 females (14 implants, three in maxilla and eleven in mandible) and 12 males (12 implants, nine in maxilla and three in mandible), with an average age of 51.8 years (33–72 years). Thirteen were placed in the FGG group (eight females, eight implants and five males, five implants) with average age of 49.8 years (37–64 years) and 13 in the Control group (six females, six implants and seven males, seven implants) with average age of 53.7 years (33–72 years)). Detailed patient characteristics are shown in Table 1.

**Table 1:** Patient characteristics

Parameters	Control group	FGG group
Mean age	53.7	49.8
Female	6 (6 implant)	8 (8 implants)
Implant sites	1 in maxilla, 5 in mandible	2 in maxilla, 6 in mandible
Male	7 (7 implants)	5 (5 implants)
Implant sites	5 in maxilla, 2 in mandible	4 in maxilla,1 in mandible)

### Clinical outcome

Detailed information on the changes in PI, GI, PD, and BOP in each group is shown in Table 2. The clinical parameters were lower in the FGG group than that in the Control group, although there were no significant differences between the two groups (PI: *P* = 0.906; GI: *P* = 0.805; PD: *P* = 0.201; BOP: *P* = 0.606). The implant rate was 100%, with no intraoperative or postoperative complications observed.

**Table 2:** Descriptive statistics (mean ± SD) of changes in peri-implant health

Parameters	Baseline	1 month	3 months	6 months	12 months	*P*
PI						
Control	1.12 ± 0.32	1.13 ± 0.43	1.19 ± 0.59	1.10 ± 0.33	1.17 ± 0.40	0.906^a^
FGG	0.63 ± 0.22	0.62 ± 0.17	0.58 ± 0.19	0.58 ± 0.16	0.56 ± 0.11	
GI						
Control	0.96 ± 0.43	0.98 ± 0.28	0.94 ± 0.29	1.04 ± 0.29	1.06 ± 0.33	0.805^a^
FGG	0.71 ± 0.29	0.69 ± 0.31	0.67 ± 0.24	0.65 ± 0.19	0.62 ± 0.19	
PD (mm)						
Control	4.2 ± 1.9	4.0 ± 1.8	3.7 ± 1.5	3.8 ± 1.9	3.3 ± 1.1	0.201^b^
FGG	3.7 ± 1.0	3.6 ± 1.3	3.5 ± 1.0	3.3 ± 1.1	3.1 ± 0.9	
BOP (%)						
Control	11.54 ± 16.51	11.57 ± 12.95	11.56 ± 12.95	9.64 ± 16.25	11.57 ± 12.94	0.606^a^
FGG	7.69 ± 12.01	5.77 ± 10.96	5.77 ± 10.96	3.85 ± 9.39	1.92 ± 6.93	

PI: plaque index; GI: gingival index; PD: probing depth; BOP: bleeding on probing;

a: results of Friedman’s two-way ANOVA by ranks. b: results of two-way ANOVA

### Peri-implant soft tissue evaluations

ΔAMM and ΔASTT values at 1, 3, 6, and 12 months after baseline are shown in Table 3. The mucosal margin receded in both groups, notably in the Control group compared to the FGG group. Significant differences were also observed between ΔAMM of the two groups at each time point (baseline-1 month: *P* = 0.002; baseline-3 months: *P* < 0.001; baseline-6 months: *P* = 0.017; baseline-12 months: *P* = 0.016). Based on the findings of ΔASTT, the soft tissue collapse was observed in both groups, but less so in the FGG group. There were significant differences between the two groups at 1 (baseline-1 month: *P* = 0.001; baseline-3 months: *P* = 0.012; baseline-6 months: *P* = 0.017; baseline-12 months: *P* = 0.044), 3 (baseline-1 month: *P* = 0.007; baseline-3 months: *P* = 0.013; baseline-6 months: *P* = 0.011; baseline-12 months: *P* = 0.016) and 5 mm below the mucosal margin (baseline-1 month: *P* = 0.013; baseline-3 months: *P* = 0.020; baseline-6 months: *P* = 0.001; baseline-12 months: *P* = 0.004).

**Table 3:** Descriptive statistics (mean ± SD, mm) of changes in peri-implant soft tissue

Parameters	1 month	3 months	6 months	12 months	Two-way ANOVA		
					Time	Group	Time × Group
ΔAMM							
Control	− 0.35± 0.18	− 0.55 ± 0.18	− 0.60 ± 0.42	− 0.61 ± 0.36	F (1,42) = 11.01	F (1,24) = 14.25	F (3,72) = 1.24
FGG	− 0.11 ± 0.10	− 0.17 ± 0.15	− 0.22 ± 0.25	− 0.31 ± 0.23	*P* < 0.001	*P* < 0.001	*P* = 0.303
*P*^c^	0.002	< 0.001	0.017	0.016	**–**	**–**	**–**
ΔASTT_1 mm_							
Control	− 0.36 ± 0.15	− 0.40 ± 0.17	− 0.52 ± 0.18	− 0.67 ± 0.28	F (2,55) = 14.13	F (1,24) = 30.65	F (3,72) = 0.67
FGG	− 0.16 ± 0.10	− 0.18 ± 0.21	− 0.23 ± 0.23	− 0.33 ± 0.21	*P* < 0.001	*P* < 0.001	*P* = 0.576
*P*^c^	0.001	0.012	0.017	0.044	**–**	**–**	**–**
ΔASTT_3 mm_							
Control	− 0.25 ± 0.12	− 0.39 ± 0.18	− 0.53 ± 0.14	− 0.67 ± 0.31	F (2,57) = 26.86	F (1,24) = 25.42	F (3,72) = 4.86
FGG	− 0.13 ± 0.08	− 0.19 ± 0.17	− 0.26 ± 0.24	− 0.32 ± 0.21	*P* < 0.001	*P* < 0.001	*P* = 0.004
*P*^c^	0.007	0.013	0.011	0.016	**–**	**–**	**–**
ΔASTT_5 mm_							
Control	− 0.20 ± 0.13	− 0.29 ± 0.18	− 0.46 ± 0.22	− 0.51 ± 0.24	F (1,36) = 45.86	F (1,24) = 14.58	F (3,72) = 9.95
FGG	− 0.08 ± 0.05	− 0.12 ± 0.08	− 0.16 ± 0.14	− 0.21 ± 0.16	*P* < 0.001	*P* < 0.001	*P* < 0.001
*P*^c^	0.013	0.020	0.001	0.004	**–**	**–**	**–**
Changes in width of KM							
Control	− 0.11 ± 0.11	− 0.21 ± 0.17	− 0.30 ± 0.13	− 0.40 ± 1.13	F (1,41) = 7.27	F (1,24) = 0.30	F (3,72) = 0.22
FGG	− 0.07 ± 0.21	− 0.15 ± 0.75	− 0.21 ± 0.58	− 0.28 ± 0.58	*P* = 0.003	*P* = 0.588	*P* = 0.882
*P*^c^	> 0.999	> 0.999	> 0.999	> 0.999	**–**	**–**	**–**

ΔAMM: changes in mucosal margin; ΔASTT: changes in soft tissue thickness; KM: keratinized mucosa. c: results of Bonferroni post-hoc test

Changes in width of KM are shown in Table 3. According to the results, significant differences of changes in width of KM were not found between the FGG and Control group (*P* > 0.999), although the width of KM was larger in the FGG group than the Control group at each time point.

## Discussion

In this study, clinical parameters and changes in peri-implant soft tissue captured using a 3Shape intraoral scanner following implant reconstruction were evaluated in groups with and without FGG before implant placement. Colorful digital models were obtained using the image processing software by transferring the format with 3Shape CAD software (Ortho Analyzer™, 3Shape, Denmark), enabling the region-of-interest to be selected easily. The results showed no significant difference in clinical parameters (PI, GI, PD, and BOP) between FGG group and Control group. Changes in mucosal margin (ΔAMM) and soft tissue thickness (ΔASTT) were minimal and were significantly greater in Control group compared to FGG group during 12 months follow-up. Changes in width of KM were minimal and showed no significant difference between the two groups. Within the limitations of this study, the null hypothesis of FGG before implant placement is beneficial to peri-implant health was not accepted. Whereas, the null hypothesis of FGG is beneficial to maintain peri-implant soft tissue was accepted.

The clinical parameters (PI, GI, PD, and BOP) were higher in Control group (fluctuation) than those in the FGG group (continuously reduction) during 12 months follow-up, although there was no significant difference between the two groups (Table 2), which suggests that the peri-implant health status in Control group was inferior to FGG group. For PI, one possible explanation is that increased KM improve the ability of patients to do plaque control [29]. In this study, results of GI were in agreement with PI suggesting that peri-implant tissue state is in line with plaque state during 12 months follow-up. For PD, this might be because KM resistance to probe increased [30] in FGG group. This increased resistance might have also affected BOP [8]. These results are in line with a retrospective case–control study demonstrating that there was no significant difference of peri-implant health in groups with or without subepithelial connective tissue graft [20]. Whereas, a systematic review concluded that soft tissue grafting for gain of keratinized mucosa result in more favorable peri-implant health [31]. In the last decade, the role of soft tissue on peri-implant health was a controversial topic [32–34]. While several researchers have shown that sufficient KM around implant is positive to maintain peri-implant health [7, 10, 35], others have reported conflicting findings [11, 36, 37]. A recent systematic review and network meta-analysis included 23 randomized controlled trials out of 52 articles reporting the outcomes of peri-implant soft tissue phenotype modification [38]. This recent study demonstrated that performing soft tissue augmentation for increasing width of KM is beneficial to improve peri-implant health (a significant reduction of probing depth and plaque index).

Collapse of the buccal soft tissue occurred after baseline (Table 3), with less changes in the FGG group during 12 months follow-up. One possible explanation for this is that the increased KM formed a more mechanically resistant and toughened surface [39] in FGG group than that in the Control group with inadequate KM. In addition, peri-implant soft tissue changes were minimal and might be considered as clinically accepted, especially in the posterior region.

In terms of changes in the mucosal margin, recessions were observed in the present study (FGG group: − 0.31 ± 0.23 mm; Control Group: − 0.61 ± 0.36 mm). This is less than a previous one-year longitudinal section study showing recession of the mucosal margin around the implant of about 1 mm [40]. One possible explanation for this is that this study performed FGG before implant placement. Moreover, the gingival biotypes which is not clear in the previous study included in this study were all medium to thick. Tian et al. evaluated the changes in peri-implant soft tissue morphology with time after immediate implant placement and restoration of the maxillary central or lateral incisor [28]. Their results revealed average mucosal recession of 0.24 ± 0.37 mm on the labial side after 1 year which is lower than this study. This might be due to the difference of implant site and implant pattern.

In this study, soft tissue collapse was observed in the two groups. ΔASTT in the FGG group was − 0.33±0.21 mm, − 0.32±0.21 mm, and − 0.21±0.16 mm at 1, 3, and 5 mm below the mucosal margin, respectively, at 12 months after baseline, while in the Control group values were − 0.67±0.28 mm, − 0.67±0.31 mm, and − 0.51±0.24 mm, respectively (Table 3). Two previous randomized controlled clinical studies reported peri-implant soft tissue thickness changes without soft tissue augmentation within a 1-year period, with collapse of − 0.15±0.20 mm (1 mm below the mucosal margin), − 0.06±0.20 mm (3 mm below the mucosal margin), and − 0.20±0.51 mm (5 mm below the mucosal margin) [41], and − 0.27±0.19 mm (1 mm below the mucosal margin), − 0.25±0.24 mm (3 mm below the mucosal margin), and − 0.33±0.32 mm (5 mm below the mucosal margin) [42], respectively. Certain procedures have subsequently been used to improve this collapse. For example, Huber et al. reported a general collapse of − 0.20±0.14 mm with a connective tissue graft performed 3 months before the second-stage procedure [21]. In the present study, ΔASTT in the FGG group were in line with those in the above previous studies, while those in the Control group were inferior. Possible explanations for this difference are the timing or category of soft tissue augmentation (FGG before implant placement) and the implant site selected in this study (posterior region). A bigger sample size is needed to confirm these findings.

In addition, although significant differences of changes in width of KM were not found between the FGG and Control group, the results for width of KM were larger in the FGG group than the Control group (Table 3), suggesting that FGG is beneficial in terms of increasing keratinized mucosa. Possible reason for this is that, in this study, FGG was performed before implant placement, and the definitive crown was inserted about 11 months after FGG. The period of graft shrinkage might have passed. For shrinkage of the graft, Lim et al. revealed that the rate of graft shrinkage was largest within 6 months after FGG (24%) [43]. A one-year prospective study investigated the changes in width of KM around implants [44]. The authors performed FGG and two-stage implant placement simultaneously after ridge augmentation and suggested that width of KM decreased 1.40 mm (30%) within one-year follow-up. Therefore, similar changes pattern in width of KM was observed in the two groups.

However, there are limitations in this study. First, the sample size was small, and tracking time was short. Second, this study only recruited posterior implant. FGG is commonly used in the posterior region because the color and texture of free gingival graft did not match well with the surrounding mucosa which limits its application in the anterior region [45]. Third, researchers give special attention to patient-reported outcome measures (PROMs) which evaluate patient satisfaction on procedures [46, 47]. Whereas, PROMs after FGG were not analyzed in this study because of limited sample size. Fourth, evaluation of clinical attachment loss and marginal bone loss were missing in that this study focuses more on peri-implant soft tissue changes. They have been used as key indicators in several studies for determining peri-implant soft and hard tissue health [8, 31, 38, 48] and should be paid close attention in the future studies. Moreover, only one type of implant system was examined. Further studies are therefore needed to address these limitations.

## Conclusions

In conclusion, minimal peri-implant soft tissue changes occurred in two groups. Performing FGG before implant placement is a viable procedure to maintain peri-implant soft tissue but might not affect peri-implant health during 12 months follow-up. Small sample size and short-term follow-up must be taken into consideration.

## Data Availability

The datasets used and analyzed during the current study are available from the corresponding author on reasonable request.

## Abbreviations

FGG: Free gingival graft
KM: Keratinized mucosa
ANOVA: Analysis of variance
PI: Plaque index
GI: Gingival index
PD: Probing depth
BOP: Bleeding on probing
VRML: Virtual reality modeling language
MM: Mucosal margin
ΔAMM: Changes in mucosal margin
STT: Soft tissue thickness
ΔASTT: Changes in soft tissue thickness
CAD: Computer aided design

## References

[1] Hu M, Chen J, Pei X, Han J, Wang J (2019). Network meta-analysis of survival rate and complications in implant-supported single crowns with different abutment materials. J Dent.

[2] Klinge B, Klinge A, Bertl K, Stavropoulos A (2018). Peri-implant diseases. Eur J Oral Sci.

[3] Lang NP, Berglundh T (2011). Peri-implant diseases: where are we now?–Consensus of the Seventh European Workshop on Periodontology. J Clin Periodontol.

[4] Sanz M, Chapple IL (2012). Clinical research on peri-implant diseases: consensus report of Working Group 4. J Clin Periodontol.

[5] Lindhe J, Meyle J (2008). Peri-implant diseases: consensus report of the Sixth European workshop on periodontology. J Clin Periodontol.

[6] Kim BS, Kim YK, Yun PY, Yi YJ, Lee HJ, Kim SG, Son JS (2009). Evaluation of peri-implant tissue response according to the presence of keratinized mucosa. Oral Surg Oral Med Oral Pathol Oral Radiol Endod.

[7] Wennström JL, Derks J (2012). Is there a need for keratinized mucosa around implants to maintain health and tissue stability?. Clin Oral Implants Res.

[8] Oh SL, Masri RM, Williams DA, Ji C, Romberg E (2017). Free gingival grafts for implants exhibiting lack of keratinized mucosa: a prospective controlled randomized clinical study. J Clin Periodontol.

[9] Buyukozdemir Askin S, Berker E, Akincibay H, Uysal S, Erman B, Tezcan İ, Karabulut E (2015). Necessity of keratinized tissues for dental implants: a clinical, immunological, and radiographic study. Clin Implant Dent Relat Res.

[10] Perussolo J, Souza AB, Matarazzo F, Oliveira RP, Araújo MG (2018). Influence of the keratinized mucosa on the stability of peri-implant tissues and brushing discomfort: a 4-year follow-up study. Clin Oral Implants Res.

[11] Monje A, Blasi G (2019). Significance of keratinized mucosa/gingiva on peri-implant and adjacent periodontal conditions in erratic maintenance compliers. J Periodontol.

[12] Zucchelli G, Tavelli L, McGuire MK, Rasperini G, Feinberg SE, Wang HL, Giannobile WV (2020). Autogenous soft tissue grafting for periodontal and peri-implant plastic surgical reconstruction. J Periodontol.

[13] Kadkhodazadeh M, Amid R, Kermani ME, Mirakhori M, Hosseinpour S (2017). Timing of soft tissue management around dental implants: a suggested protocol. Gen Dent.

[14] Bengazi F, Lang NP, Caroprese M, Urbizo Velez J, Favero V, Botticelli D (2015). Dimensional changes in soft tissues around dental implants following free gingival grafting: an experimental study in dogs. Clin Oral Implants Res.

[15] Imano MH, Cunha EJ, Storrer CLM, Deliberador TM (2019). A modified free gingival graft technique for gaining vertical and horizontal soft tissue augmentation. J Indian Soc Periodontol.

[16] Rojo E, Stroppa G, Sanz-Martin I, Gonzalez-Martín O, Alemany AS, Nart J: Soft tissue volume gain around dental implants using autogenous subepithelial connective tissue grafts harvested from the lateral palate or tuberosity area. A randomized controlled clinical study. *J Clin Periodontol* 2018, 45(4):495–503. 10.1111/jcpe.12869 29334403

[17] Fageeh HN, Meshni AA, Jamal HA, Preethanath RS, Halboub E (2019). The accuracy and reliability of digital measurements of gingival recession versus conventional methods. BMC Oral Health.

[18] Tavelli L, Barootchi S, Majzoub J, Siqueira R, Mendonça G, Wang HL (2021). Volumetric changes at implant sites: A systematic appraisal of traditional methods and optical scanning-based digital technologies. J Clin Periodontol.

[19] Schneider D, Grunder U, Ender A, Hammerle CH, Jung RE (2011). Volume gain and stability of peri-implant tissue following bone and soft tissue augmentation: 1-year results from a prospective cohort study. Clin Oral Implants Res.

[20] Bienz SP, Jung RE, Sapata VM, Hammerle CHF, Husler J, Thoma DS (2017). Volumetric changes and peri-implant health at implant sites with or without soft tissue grafting in the esthetic zone, a retrospective case-control study with a 5-year follow-up. Clin Oral Implants Res.

[21] Huber S, Zeltner M, Hämmerle CHF, Jung RE, Thoma DS (2018). Non-interventional 1-year follow-up study of peri-implant soft tissues following previous soft tissue augmentation and crown insertion in single-tooth gaps. J Clin Periodontol.

[22] Thoma DS, Gasser TJW, Jung RE, Hämmerle CHF (2020). Randomized controlled clinical trial comparing implant sites augmented with a volume-stable collagen matrix or an autogenous connective tissue graft: 3-year data after insertion of reconstructions. J Clin Periodontol.

[23] Bergendal B (2008). When should we extract deciduous teeth and place implants in young individuals with tooth agenesis?. J Oral Rehabil.

[24] Fischer KR, Künzlberger A, Donos N, Fickl S, Friedmann A (2018). Gingival biotype revisited-novel classification and assessment tool. Clin Oral Investig.

[25] de Siqueira RAC, Fontão F, Sartori IAM, Santos PGF, Bernardes SR, Tiossi R (2017). Effect of different implant placement depths on crestal bone levels and soft tissue behavior: a randomized clinical trial. Clin Oral Implants Res.

[26] Silness J, Loe H (1964). Periodontal disease in pregnancy. II. Correlation between oral hygiene and periodontal condtion. Acta Odontol Scand.

[27] Löe H (1967). The gingival index, the plaque index and the retention index systems. J Periodontol.

[28] Tian J, Wei D, Zhao Y, Di P, Jiang X, Lin Y (2019). Labial soft tissue contour dynamics following immediate implants and immediate provisionalization of single maxillary incisors: a 1-year prospective study. Clin Implant Dent Relat Res.

[29] Souza AB, Tormena M, Matarazzo F, Araújo MG (2016). The influence of peri-implant keratinized mucosa on brushing discomfort and peri-implant tissue health. Clin Oral Implants Res.

[30] Vitek RM, Robinson PJ, Lautenschlager EP (1979). Development of a force controlled periodontal probing instrument. J Periodontal Res.

[31] Thoma DS, Naenni N, Figuero E, Hämmerle CHF, Schwarz F, Jung RE, Sanz-Sánchez I (2018). Effects of soft tissue augmentation procedures on peri-implant health or disease: a systematic review and meta-analysis. Clin Oral Implants Res.

[32] Thoma DS, Mühlemann S, Jung RE (2014). Critical soft-tissue dimensions with dental implants and treatment concepts. Periodontol 2000.

[33] Cairo F, Pagliaro U, Nieri M (2008). Soft tissue management at implant sites. J Clin Periodontol.

[34] Giannobile WV, Jung RE, Schwarz F (2018). Evidence-based knowledge on the aesthetics and maintenance of peri-implant soft tissues: osteology foundation consensus report part 1-effects of soft tissue augmentation procedures on the maintenance of peri-implant soft tissue health. Clin Oral Implants Res.

[35] Schou S, Holmstrup P, Hjørting-Hansen E, Lang NP (1992). Plaque-induced marginal tissue reactions of osseointegrated oral implants: a review of the literature. Clin Oral Implants Res.

[36] Grischke J, Karch A, Wenzlaff A, Foitzik MM, Stiesch M, Eberhard J (2019). Keratinized mucosa width is associated with severity of peri-implant mucositis. A cross-sectional study. Clin Oral Implants Res.

[37] Bouri A, Bissada N, Al-Zahrani MS, Faddoul F, Nouneh I (2008). Width of keratinized gingiva and the health status of the supporting tissues around dental implants. Int J Oral Maxillofac Implants.

[38] Tavelli L, Barootchi S, Avila-Ortiz G, Urban IA, Giannobile WV, Wang HL (2021). Peri-implant soft tissue phenotype modification and its impact on peri-implant health: a systematic review and network meta-analysis. J Periodontol.

[39] Groeger SE, Meyle J (2015). Epithelial barrier and oral bacterial infection. Periodontol 2000.

[40] Small PN, Tarnow DP (2000). Gingival recession around implants: a 1-year longitudinal prospective study. Int J Oral Maxillofac Implants.

[41] Sanz Martin I, Benic GI, Hammerle CH, Thoma DS (2016). Prospective randomized controlled clinical study comparing two dental implant types: volumetric soft tissue changes at 1 year of loading. Clin Oral Implants Res.

[42] Sapata VM, Sanz-Martin I, Hammerle CHF, Cesar Neto JB, Jung RE, Thoma DS (2018). Profilometric changes of peri-implant tissues over 5 years: a randomized controlled trial comparing a one- and two-piece implant system. Clin Oral Implants Res.

[43] Lim HC, An SC, Lee DW (2018). A retrospective comparison of three modalities for vestibuloplasty in the posterior mandible: apically positioned flap only vs. free gingival graft vs. collagen matrix. Clin Oral Investig.

[44] Stimmelmayr M, Stangl M, Edelhoff D, Beuer F (2011). Clinical prospective study of a modified technique to extend the keratinized gingiva around implants in combination with ridge augmentation: one-year results. Int J Oral Maxillofac Implants.

[45] Agarwal C, Tarun Kumar AB, Mehta DS (2015). Comparative evaluation of free gingival graft and AlloDerm(®) in enhancing the width of attached gingival: a clinical study. Contemp Clin Dent.

[46] Tavelli L, Barootchi S, Di Gianfilippo R, Kneifati A, Majzoub J, Stefanini M, Zucchelli G, Wang HL (2021). Patient experience of autogenous soft tissue grafting has an implication for future treatment: A 10- to 15-year cross-sectional study. J Periodontol.

[47] Di Gianfilippo R, Wang IC, Steigmann L, Velasquez D, Wang HL, Chan HL: Efficacy of microsurgery and comparison to macrosurgery for gingival recession treatment: a systematic review with meta-analysis. Clin Oral Investig. 2021; 10.1007/s00784-021-03954-0 33928441

[48] Lorenzo R, García V, Orsini M, Martin C, Sanz M (2012). Clinical efficacy of a xenogeneic collagen matrix in augmenting keratinized mucosa around implants: a randomized controlled prospective clinical trial. Clin Oral Implants Res.

